# Prolonged Post-Scabietic Pruritus: Clinical Characteristics and Unmet Management Needs in a Real-World Cohort

**DOI:** 10.3390/jcm15093211

**Published:** 2026-04-23

**Authors:** Özlem Akın, Mehmet Oktay Taşkapan, Özlem Tanrıöver

**Affiliations:** 1 Department of Dermatology, Faculty of Medicine, Yeditepe University, Istanbul 34752, Türkiye; oktaytaskapan@hotmail.com; 2 Department of Medical Education, Faculty of Medicine, Marmara University, Istanbul 34752, Türkiye; drozlemtan56@gmail.com

**Keywords:** post-scabietic pruritus, scabies, chronic pruritus, nodular lesions, neurogenic itch

## Abstract

**Background:** Scabies is a highly contagious ectoparasitic skin infection that is considered a global public health problem. Treatment primarily focuses on mite eradication, but an inflammatory phase may persist despite successful therapy, known as post-scabietic pruritus (PSP). This study aimed to investigate the clinical features, duration, and severity of PSP in patients with persistent symptoms despite successful treatment. **Methods:** This cross-sectional descriptive study included 43 adult patients with dermoscopically documented scabies resolution, who reported pruritus persisting for more than 28 days. Demographic and clinical data, including clinical characteristics, treatment history, and pruritus severity assessed by the Visual Analogue Scale (VAS), were collected via structured questionnaires. **Results:** PSP persisted for 30–365 days, and 48.8% of patients experienced pruritus longer than 45 days. Pruritus severity in 72.1% of patients was reported as a VAS score ≥8. Nodular lesions were observed in 55.8% of patients. Sixteen patients (37.2%) had an atopic background, and nine patients (20.9%) reported a house dust mite (HDM) allergy. Heat, hot baths, and sweating were the most frequent aggravating factors. Despite persistent symptoms, 44.2% of patients reported not receiving any specific treatment after mite eradication. **Conclusions:** PSP can be prolonged and severe. Nodular lesions may be more frequent than previously reported. Eradication of the mite is the first step, and treatment is not complete until the post-treatment inflammatory phase is controlled.

## 1. Introduction

Scabies is caused by the ectoparasite *Sarcoptes scabiei* var. *hominis* and affects more than 200 million people worldwide annually [1]. Although scabies is a treatable condition, it is still a leading cause of skin-related morbidity, particularly in tropical regions within East Asia, Southeast Asia, Oceania, and tropical Latin America, especially in children, adolescents, and the elderly due to evolving resistance patterns and delayed diagnosis [2,3,4]. Its most burdensome symptom is pruritus, which typically intensifies at night, and is caused by an immediate or delayed (type IV) hypersensitivity reaction to the mite, its eggs, and its fecal pellets (scybala) [1,5].

While international guidelines provide effective treatment protocols for the eradication of the mite, management of the period following successful treatment is poorly defined [3,5]. Post-scabietic pruritus (PSP) is used to describe itching that persists after biological resolution of the infestation [6]. PSP is commonly described in the literature as a self-limiting phase lasting approximately two to four weeks [7]. However, the available data on this issue remain limited, and some studies suggest that PSP may persist longer than traditionally described [6,7,8]. Although post-scabietic pruritus has been reported after successful scabies treatment, its true prevalence remains uncertain, as most studies describe it descriptively rather than quantitatively.

Persistent scabies-related itching can lead to psychological consequences. Patients who continue to itch after treatment may suffer from scabies phobia or delusional parasitosis, believing the treatment has failed. This can lead to repeated applications of scabicides, which further compromise the skin barrier and exacerbate pruritus [6,7]. Therefore, determination of the clinical features of PSP is essential for both the patient’s quality of life and rational drug use.

This study aims to analyze the clinical profile of PSP and identify therapeutic gaps in its current management.

## 2. Materials and Methods

### 2.1. Study Design and Setting

This cross-sectional descriptive study was conducted at a tertiary dermatology outpatient clinic. Patient identification and eligibility assessment were based on clinical records from 2021 to 2025, while data collection was carried out between January and February 2026. Although patient identification was based on retrospective clinical records, data collection was performed at a single time point; therefore, the study is best considered a cross-sectional study with retrospective elements. Patients were contacted after confirmation of treatment response documented in their medical records.

### 2.2. Study Population

Between 2021 and 2025, 340 adult patients (≥18 years) with dermoscopically (DermLite DL5, 3Gen Inc., San Juan Capistrano, CA, USA) confirmed scabies who subsequently demonstrated dermoscopic resolution following appropriate scabicidal treatment were identified.

Dermoscopic resolution after treatment was documented in the patients’ medical records; however, dermoscopic re-evaluation was not routinely performed immediately prior to study participation. Eligibility was based on dermoscopically confirmed treatment response and the absence of clinical features suggestive of active infestation at the time of contact; patients with suspected treatment failure or reinfestation were excluded.

These patients constituted the initial study pool. Patients were then screened for persistent symptoms, and those reporting pruritus lasting longer than 4 weeks (>28 days) after completion of treatment were considered eligible for inclusion. Patients with clinical findings or medical history suggestive of other dermatologic or systemic causes of chronic pruritus were excluded based on clinical evaluation and review of medical records.

### 2.3. Data Collection

Eligible patients were contacted during the study period (January–February 2026) and invited to participate. Data were collected using a structured questionnaire administered at a single time point. The questionnaire was developed by the authors based on clinical experience and relevant studies. Although it was not formally validated, it was reviewed for clarity and comprehensibility prior to use. Only patients who provided written informed consent and fully completed the questionnaire were included in the final analysis.

Collected variables included sociodemographic characteristics, medical history and comorbidities, scabies history and treatment characteristics, pruritus characteristics (onset, duration, severity, and distribution), aggravating factors, presence of nodular pruritic lesions, and post-treatment management strategies. Systemic medication use was defined as the use of any ongoing systemic medications for comorbid conditions at the time of study participation.

Pruritus severity was assessed using the Visual Analogue Scale (VAS; 0–10).

In addition, post-treatment management practices, including physician-prescribed therapies and patient-initiated measures, were specifically evaluated to identify potential therapeutic gaps.

Patients who did not report persistent pruritus, declined participation, or did not complete the questionnaire were excluded from the analysis. As participation was voluntary and data were collected via an online questionnaire, selection bias cannot be excluded.

### 2.4. Ethical Considerations

This study was approved by the Institutional Ethics Committee (approval number: 09.2026.26-00046; approval date: 20 January 2026) and was conducted in accordance with the principles of the Declaration of Helsinki. Written informed consent was obtained from all participants prior to questionnaire completion in 2026. Retrospective clinical records (2021–2025) were used solely for patient identification and eligibility assessment in accordance with ethical approval. The study involved no invasive procedures, and all data were anonymized and stored securely in accordance with institutional policies.

### 2.5. Statistical Analysis

Statistical analyses were performed using IBM SPSS Statistics version 25.0 (IBM Corp., Armonk, NY, USA). The normality of continuous variables was assessed using the Shapiro–Wilk test and visual inspection of histograms and Q–Q plots. Continuous variables were expressed as median [IQR]. Categorical variables were presented as number (percentage). Comparisons between groups with severe PSP (VAS ≥ 8) and non-severe pruritus (VAS < 8) were performed using the Chi-square test or Fisher’s exact test, as appropriate. Similarly, factors associated with prolonged PSP (≥45 days) were evaluated using Chi-square or Fisher’s exact tests. For exploratory stratification of symptom persistence beyond the minimum inclusion threshold of 28 days, prolonged PSP was operationally defined as ≥45 days. This data-driven threshold corresponded to the median post-treatment pruritus duration observed in our cohort and allowed for a balanced comparison between shorter and more sustained symptom persistence. Analyses were primarily descriptive, reflecting the study’s exploratory nature. A two-sided *p*-value < 0.05 was considered statistically significant. No formal sample size calculation was performed, as this was an exploratory study including all eligible patients who met the inclusion criteria during the study period. Given the relatively small sample size and the exploratory nature of the study, multivariable modeling was not performed to avoid overfitting and unstable estimates. The analyses were therefore intentionally restricted to univariate comparisons. This study was conducted and reported in accordance with the Strengthening the Reporting of Observational Studies in Epidemiology (STROBE) guidelines.

## 3. Results

**Figure 1:**
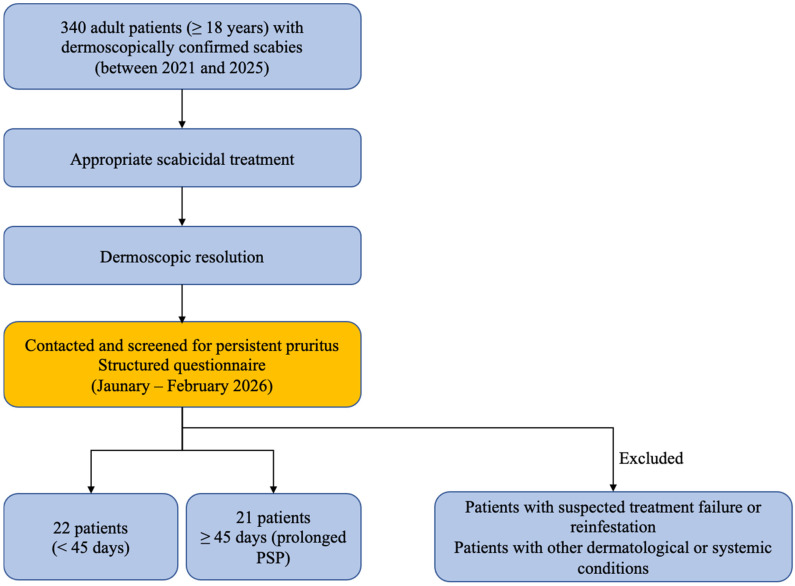
Flow diagram illustrating the patient selection process, including identification, eligibility assessment, and inclusion of patients with prolonged post-scabietic pruritus.

Among the 340 patients initially screened, 43 (12.6%) met the inclusion criteria for prolonged post-scabietic symptoms, defined as pruritus persisting for more than 28 days despite dermoscopic confirmation of parasite eradication. The patient selection process is illustrated in Figure 1. The baseline demographic and clinical characteristics of the study population are summarized in Table 1. Patients were predominantly young adults, with a slight male predominance. More than half of the patients presented with nodular lesions, a considerable proportion (16/43, 37.2%) had an atopic background, and most patients (36/43, 83.7%) reported at least one aggravating factor.

Systemic comorbidities were predominantly cardiovascular, endocrine/metabolic, and neurological/psychiatric disorders. Similarly, commonly reported medications included antihypertensives, antidiabetics, and psychiatric agents. These variables were not significantly associated with study outcomes.

A considerable proportion of patients (31/43, 72.1%) adopted self-management strategies, and nearly half of the patients (19/43, 44.2%) received no specific treatment after mite eradication (Table 2).

Pruritus was severe (VAS ≥ 8 in 31/43, 72.1%) and often prolonged, with a substantial proportion of patients reporting persistence beyond six weeks (Table 3).

As shown in Table 4, patients with severe PSP were more likely to have received treatment compared with those with non-severe symptoms (67.7% vs. 25.0%, *p* = 0.017). Neither nodular lesions nor allergic disease/atopy were significantly associated with severe pruritus.

Regarding prolonged PSP (≥45 days), neither nodular lesions nor allergic disease/atopy were significantly associated with symptom duration (*p* = 0.763 and *p* = 0.755, respectively) (Table 5).

Most patients (31/43, 72.1%) reported adopting personal measures to relieve pruritus. The most commonly reported practices were the use of sulfur-containing products (16 patients, 37.2%) and frequent bathing or cold/lukewarm showers (12 patients, 27.9%). Vinegar-based solutions and cologne or alcohol-based topical agents were each reported by eight patients (18.6%). Herbal products and over-the-counter anti-pruritic creams were rarely used (one patient each, 2.3%).

## 4. Discussion

Our study demonstrated that PSP is not only a transient sequel but could be a prolonged and severe clinical entity. To our knowledge, this is one of the few studies specifically focusing on prolonged post-scabietic pruritus beyond the traditionally described 2–4 week period, and among the first to systematically explore its clinical severity, nodular presentation, and management gaps in a real-world cohort. While the literature typically suggests that post-treatment pruritus resolves within 2–4 weeks, our findings, consistent with a limited number of previous reports, show persistence for up to 1 year in a subset of patients [6,7,8].

The ≥45-day threshold used to define prolonged post-scabietic pruritus was based on the median symptom duration observed in our cohort and should be considered a pragmatic, data-driven cutoff. Currently, there is no standardized definition for prolonged PSP in the literature. Therefore, this classification should be interpreted as exploratory rather than a validated clinical definition.

In our study, nearly half of the patients (44.2%) remained untreated despite persistent pruritus, highlighting a significant post-scabietic management gap. This suggests that PSP may be underestimated or misinterpreted in clinical practice, leaving patients to cope with avoidable distress.

### 4.1. Severity and Chronicity of Symptoms

One of the most notable observations in our study was that 72.1% of patients reported VAS scores of 8 or higher, indicating that PSP often manifests as severe pruritus. With symptom duration ranging from 30 to 365 days, our findings suggest that PSP does not always resolve within the few weeks typically described in the literature. This prolonged course may be partially explained by mechanisms related to Type IV (delayed-type) hypersensitivity. Scabicidal agents kill the mite but do not remove the residual antigenic material. Residual mite antigens embedded in the stratum corneum act as a persistent stimulus for T-cell activation, leading to the release of itch-related cytokines such as IL-31 (Interleukin-31) and contributing to ongoing pruritus. Scabies may begin with a predominantly T helper 1 (Th1)-mediated immune response; however, prolonged post-treatment symptoms could be linked to a subsequent shift toward Th2 activity [9]. These proposed mechanisms are hypothetical and based on previous research, as they were not directly assessed in our study.

### 4.2. Nodular Subtype

More than half of the patients in our study (55.8%) presented with nodular lesions. This rate is higher than the 7–10% typically reported in the general scabies population [10]. However, this finding should be interpreted with caution, as our cohort specifically included patients with prolonged post-treatment symptoms rather than unselected scabies cases. Therefore, the observed frequency may reflect the selection bias of the study population rather than the true prevalence among all scabies patients.

Post-scabietic nodules have been described as a manifestation of a prolonged hypersensitivity reaction to residual mite antigens and may persist for several months or longer. These lesions can present as nodular or papular eruptions and may resemble nodular scabies, making accurate clinical distinction essential to avoid unnecessary retreatment [11,12,13,14]. In our study, nodular lesions were more frequent among patients with severe pruritus; however, this association did not reach statistical significance. Given the limited sample size and cross-sectional design, the study may have been underpowered to detect such differences. Overall, nodular lesions may contribute to persistent symptoms, but their exact role remains uncertain.

### 4.3. Environmental Aggravating Factors

In our study, 83.7% of patients reported that pruritus intensity was exacerbated by heat and sweating. These factors are known to exacerbate various forms of pruritus by increasing skin blood flow and lowering the itch threshold. Persistent itching may lower the activation threshold of cutaneous C-fibers, increasing sensitivity to physical stimuli. This process, representing the transition from “inflammatory itch” to “neurogenic itch,” explains why antihistamines are often ineffective, as these drugs primarily target histamine-mediated pathways [9,15]. Consequently, management of these patients requires a shift towards calming the neural system or using potent anti-inflammatory agents rather than antiparasitic treatments. Furthermore, educating patients to avoid these triggers should be a standard component of post-scabietic management.

### 4.4. Atopic Predisposition and House Dust Mite Allergy

In our study, 16 patients reported an atopic predisposition with a history of atopic dermatitis and/or respiratory allergic disease, and 9 patients (20.9%) reported a documented HDM allergy. In the general adult population, the prevalence of atopic dermatitis is typically reported at approximately 2–17%, and of allergic rhinitis at 10–30%, both in Türkiye and globally [16,17,18]. Similarly, HDM sensitization rates in unselected adult populations generally range between 10% and 20%, depending on geographic and environmental factors [17,19]. Although not statistically compared with a matched control population, the observed prevalence appears to be at the upper range of reported population estimates. The relatively high rate of atopic background in our patients may be relevant to the persistence and severity of PSP. Atopic individuals have a lower itch threshold, impaired epidermal barrier function, and a Th2 immune response, all of which may predispose to prolonged inflammatory and neurogenic itch following successful scabies eradication [9,20]. Both scabies and atopic dermatitis are characterized by a Th2 immune response, suggesting that scabies may represent a transient atopic dermatitis-like state driven by hypersensitivity to mite antigens [21]. A previous study demonstrated that antigens of Sarcoptes scabiei cross-react with antigens of the HDM Dermatophagoides pteronyssinus [22]. In another study, a significantly higher prevalence of HDM sensitivity was found among patients with scabies, suggesting that the antigenic similarity between these two mite species could be responsible for prolonged post-treatment pruritus [21]. These findings suggest that atopic predisposition may amplify PSP severity rather than act as a causal factor. These associations should be interpreted cautiously, as the descriptive design of the present study does not allow for causal inference.

In our cohort, we did not observe a clear association between PSP and systemic comorbidities, a prior history of pruritic dermatoses, or pre-diagnostic fear of scabies transmission. However, due to the small sample size, these findings should be interpreted with caution. The study may have been underpowered to detect subtle associations, and, therefore, the relationship between these factors and PSP remains uncertain.

### 4.5. Clinical Implications and Management Gaps

A critical finding of our study is that nearly half of the patients were left untreated. In routine practice, disappearance of burrows is often interpreted as complete recovery. However, eradication of the mite does not immediately terminate the immune response it has initiated [6,7,8]. When PSP is not recognized as a distinct post-treatment phase, patients may undergo unnecessary repeated courses of scabicidal therapy, further impairing the skin barrier and perpetuating pruritus [6,7]. Persistent itch may also contribute to anxiety and depressive symptoms, and in some individuals, reinforce fears of ongoing infestation [23,24]. Interestingly, patients with severe PSP were more likely to have received treatment. This association should not be interpreted as a causal relationship. Rather, it likely reflects treatment-seeking behavior, with patients experiencing greater symptom burden being more inclined to seek or receive medical care.

Management of PSP should therefore begin with confirmation of successful mite eradication [5,6]. Once active infestation is excluded, treatment should focus on controlling residual inflammatory and neurogenic itch. A stepwise approach may be considered in clinical practice. First, in mild to moderate cases, short-term topical corticosteroids and regular emollient use are usually sufficient [6,7]. Second, antihistamines may be used for symptomatic relief, although their benefit may be limited in non-histaminergic pathways [9,15]. Finally, in more severe or nodular cases, potent topical corticosteroids, intralesional corticosteroids, or topical calcineurin inhibitors may be required [11,14]. Clear patient education regarding the inflammatory nature and potential duration of PSP is essential to prevent overtreatment and reduce psychological distress.

One of the primary strengths of our study is that all patients were diagnosed and confirmed to have achieved clinical resolution by dermoscopic examination, which eliminated the risk of including patients with active infestation in the PSP cohort. Secondly, unlike many studies that focus only on the acute phase of scabies, this research provides a detailed account of the long-term clinical course, documenting symptoms that persist for up to 365 days. Thirdly, our study identifies a significant and previously under-reported clinical gap, revealing that approximately one-third (32.3%) of patients with severe symptoms (VAS ≥ 8) remained untreated after parasite eradication. Finally, our study was conducted in a single tertiary dermatology center, ensuring consistency in diagnostic criteria and treatment protocols during the initial scabies management phase.

While our study provides important insights into the clinical characteristics of PSP, several limitations should be considered. First, the use of a non-validated questionnaire represents a methodological limitation. Although the questionnaire was developed based on clinical experience and relevant research and reviewed for clarity, the lack of formal validation may affect the reliability and reproducibility of the collected data. Furthermore, as data were collected retrospectively based on patient recall, recall bias cannot be excluded, particularly with respect to symptom duration and severity. Patients with more severe or persistent symptoms may have been more likely to recall and report their experiences, potentially leading to overestimation.

Second, the cross-sectional design precludes causal inferences and introduces potential selection bias. Participation was voluntary, and individuals with ongoing or more severe symptoms may have been more likely to participate, which may limit the generalizability of the findings.

Third, the relatively small sample size may have limited the statistical power to detect potential associations. In addition, multivariable analysis was not performed due to the limited number of events, and the findings should therefore be interpreted as exploratory.

Finally, the absence of a control group of patients without persistent pruritus limits the ability to identify independent risk factors for prolonged symptoms. Moreover, post-treatment irritant or allergic contact dermatitis was not systematically evaluated and may have contributed to persistent pruritus in some patients.

## 5. Conclusions

Prolonged post-scabietic pruritus may represent an under-recognized inflammatory phase after successful scabies eradication, often presenting with severe itch and nodular lesions and frequently remaining untreated in routine clinical practice.

## Figures and Tables

**Table 1:** Demographic and clinical characteristics of participants (n = 43).

Variable	n (%)
**Demographic characteristics**	
Age (years), Median [IQR]	37 (26–52)
Male	25 (58.1%)
Married	20 (46.5%)
**Allergic and clinical characteristics**	
Allergic disease/atopy	16 (37.2%)
House dust mite allergy	9 (20.9%)
Nodules present	24 (55.8%)
≥2 scabies episodes	7 (16.3%)
**Aggravating factors for pruritus**	
≥1 aggravating factor *	36 (83.7%)
No aggravating factor reported	7 (16.3%)
**Systemic comorbidity**	
None	27 (62.8%)
≥1 systemic comorbidity	16 (37.2%)
Immunosuppressive disease or drug use	5 (11.6%)
**Systemic medication use**	
None	26 (60.5%)
One medication	4 (9.3%)
Two or more medications	13 (30.2%)

* Aggravating factors included heat exposure, hot baths, and sweating.

**Table 2:** Patient perceptions, self-management behaviors, and treatment after scabies eradication (n = 43).

Variable	n (%)
History of pruritic dermatosis before scabies	18 (41.9%)
Fear of contracting scabies before diagnosis	5 (11.6%)
Adopted personal measures to relieve pruritus *	31 (72.1%)
No treatment for persistent pruritus after mite eradication	19 (44.2%)
Received treatment for persistent pruritus **	24 (55.8%)

* Personal measures include self-initiated practices such as sulfur-containing products, frequent bathing, vinegar-based solutions, or alcohol-containing topical applications. ** Treatment refers to physician-prescribed anti-inflammatory or anti-pruritic therapies initiated after confirmed mite eradication.

**Table 3:** Severity and duration of pruritus before diagnosis and after scabies treatment.

Outcome	Median [IQR]
Pruritus severity (VAS, 0–10)	9 (7–10)
Pre-diagnosis pruritus duration (days)	30 (14–90)
Post-treatment pruritus duration (days)	45 (30–90)

VAS: Visual Analogue Scale (0 = no itch, 10 = worst imaginable itch). IQR: Interquartile range. Duration variables are expressed in days.

**Table 4:** Factors associated with severe post-scabietic pruritus (PSP). (VAS < 8, n = 12; VAS ≥ 8, n = 31.)

Variable	Non-Severe (VAS < 8) n (%)	Severe (VAS ≥ 8) n (%)	*p*-Value
Treatment received	3 (25.0%)	21 (67.7%)	0.017
Nodular lesions	8 (66.6%)	16 (51.6%)	0.500
Allergic disease/atopy	3 (25.0%)	13 (41.9%)	0.484

Severe PSP was defined as VAS ≥ 8. Comparisons were performed using Chi-square or Fisher’s exact test, based on expected frequencies. PSP: Post-scabietic pruritus; VAS: Visual Analogue Scale.

**Table 5:** Factors associated with prolonged post-scabietic pruritus (PSP). (Duration < 45 days n = 22; ≥45 days n = 21.)

Variable	<45 Days n (%)	≥45 Days n (%)	*p*-Value
Nodular lesions	13 (59.1%)	11 (52.4%)	0.763
Allergic disease/atopy	9 (40.9%)	7 (33.3%)	0.755

Prolonged PSP was defined as pruritus lasting ≥45 days after completion of scabicidal treatment. Comparisons were performed using Chi-square or Fisher’s exact test based on expected frequencies. PSP: Post-scabietic pruritus.

## Data Availability

The datasets generated and/or analyzed during the current study are available from the corresponding author on reasonable request.

## Abbreviations

PSP	post-scabietic pruritus;
VAS	Visual Analogue Scale;
HDM	house dust mite
